# Effects of a step-by-step inpatient rehabilitation program on self-care ability and quality of life in patients with acute cerebral infarction following intravascular stent implantation: a prospective cohort study

**DOI:** 10.3389/fneur.2024.1400437

**Published:** 2024-05-01

**Authors:** Chen Wei, Nannan Xi, Jieqiong Tang, Qiangqiang Chu, Qingquan Bi

**Affiliations:** ^1^School of Nursing, Anhui Medical University, Hefei, Anhui Province, China; ^2^The Third Affiliated Hospital of Anhui Medical University, Hefei, Anhui Province, China; ^3^The Second Affiliated Hospital of Anhui University of Traditional Chinese Medicine, Hefei, Anhui Province, China

**Keywords:** self-care ability, quality of life, acute cerebral infarction, intravascular stent implantation, step-by-step inpatient rehabilitation

## Abstract

**Objective:**

This study aims to evaluate the influence of a step-by-step inpatient rehabilitation program (SIRP) on the self-care capability and quality of life of patients who have undergone intravascular stent implantation to treat large vessel occlusion during acute cerebral infarction (ACI).

**Methods:**

This study included a cohort of 90 patients with ACI who received intravascular stent implantations at a tertiary hospital in the Third Affiliated Hospital of Anhui Medical University from January 2020 to February 2024. The patients were followed up for at least 3 months. Cohort grouping was based on the type of nursing care each patient received. The observation group participated in SIRP along with receiving routine nursing care, whereas the control group received only routine nursing care. Key outcome measures included the Barthel index, the National Institute of Health Stroke Scale (NIHSS) score, the incidence of complications, length of hospital stay, and 36-item short-form survey (SF-36) scores. These parameters were compared between the two groups.

**Results:**

At the time of admission, there were no significant differences in demographic data, NIHSS score, Barthel index, or SF-36 scores between the observation and control groups (all *p* > 0.05). However, at 3 months postoperatively, the observation group showed significant improvements, with higher average scores in the Barthel index (62.49 ± 7.32 vs. 53.16 ± 4.37, *p* < 0.001) and SF-36 scores (502.33 ± 14.28 vs. 417.64 ± 9.65, *p* < 0.001). Additionally, this group had significantly lower NIHSS scores (3.38 ± 1.19 vs. 10.24 ± 2.10, *p* < 0.001), fewer complications (3 vs. 15, *p* = 0.002), and shorter hospital stays (12.40 ± 1.68 vs. 15.56 ± 1.87, *p* < 0.001).

**Conclusion:**

Implementing SIRP notably enhanced self-care capabilities and overall quality of life, while also reducing complication rates and the length of hospital stays for patients with ACI who underwent intravascular stent implantation. This underscores the potential benefits of incorporating structured rehabilitation programs in the treatment and recovery processes of such patients.

## 1 Introduction

Ischemic stroke, also known as acute cerebral infarction (ACI), is associated with significant morbidity, mortality, and disability rates, making it a leading cause of health issues and fatalities in the adult population worldwide (1). National stroke surveys indicate that the incidence, disability, and mortality rates of stroke stand at 0.25, 1.11, and 0.12%, respectively, highlighting the profound impact of ischemic stroke on the health and quality of life of individuals, as well as the substantial burden it imposes on families and society (2). Additionally, a previous study has shown that stroke can manifest as an initial symptom of certain hematological disorders or as a complication of such conditions (3). Accurate identification of underlying hematological diseases is crucial for the prompt and appropriate treatment of patients with stroke. In clinical practice, interventions such as intravenous thrombolysis, intra-arterial thrombectomy, and intravascular stent implantation are recognized as effective treatments for ischemic stroke (4). The therapeutic window for intravenous thrombolysis is restricted to 6 h, while arterial thrombectomy can be performed within a 24-h window, thus extending the opportunity for treatment (5). Intravascular stent implantation alleviates luminal stenosis by placing a stent that covers the torn section of the arterial intima, helps prevent thrombus formation, and effectively stops the progression of ischemic penumbra into full infarction or nerve cell necrosis (6).

Despite advancements in the diagnosis, management, and critical care for patients with ACI, stroke-related complications continue to significantly threaten patient survival and quality of life (7). Patients who have undergone intravascular stent implantation are particularly susceptible to complications such as intracranial hemorrhage, reperfusion injury, pneumonia, pressure ulcers, urinary tract infections, and other adverse events. These complications are often exacerbated by prolonged bed rest and compromised immunity, which can hinder recovery and lead to fatal outcomes (8). Consequently, providing high-quality, efficient, and standardized nursing care after intravascular stent implantation is crucial for optimizing patient outcomes.

The step-by-step inpatient rehabilitation program (SIRP) is a progressive training approach that aims to enhance specific skills or behaviors through gradual, continuous, and repetitive training sessions (9). SIRP is designed to increase the adaptability and resilience of the body, boost learning efficiency, and ultimately help restore near-normal functional capacity in patients (10). However, the effectiveness of SIRP on rehabilitation outcomes for patients with stroke after intravascular stent implantation has not been thoroughly investigated.

This study introduces a structured SIRP for patients with ACI who have undergone intravascular stent implantation. The program is divided into five phases and includes preoperative preparation, postoperative care, and long-term follow-up. It employs a collaborative nursing approach that involves physicians, nurses, patients, and family members. The hypothesis is that patients participating in this step-by-step program will show an improved quality of life after rehabilitation, up to 3 months, compared to those undergoing conventional rehabilitation. The study aims to evaluate the effectiveness of SIRP in enhancing rehabilitation outcomes and determine its advantages over routine inpatient rehabilitation in terms of improvements in self-care abilities, quality of life, complication rates, and length of hospital stays.

## 2 Patients and methods

### 2.1 Participants

This study is a prospective cohort investigation conducted at the Third Affiliated Hospital of Anhui Medical University. It included a total of 90 patients diagnosed with ACI who underwent interventional intravascular stent implantation treatment between January 2020 and February 2024. These patients were divided into observation and control groups based on different nursing patterns. All participants met the criteria specified in the 2018 edition of the Chinese Guidelines for the Diagnosis and Treatment of Acute Ischemic Stroke (11). The inclusion criteria were as follows: (i) patients within 24 h of symptom onset and deemed suitable for intravascular stent implantation; and (ii) informed consent provided by either the patients themselves or their representatives. The exclusion criteria included (i) severe cardiac, pulmonary, hepatic, and renal insufficiencies; and (ii) failure during the intraoperative intravascular stent implantation process. Initially, 106 patients were eligible; however, exclusions were made for five cases of surgical failure and 11 cases of postoperative mortality within 3 months [causes of death included sudden cardiac death (*n* = 2), multiorgan failure (*n* = 2), reinfarctions (*n* = 2), intracranial hemorrhage (*n* = 3), and other causes (*n* = 2); total mortality rate: 10.4%]. This resulted in a final cohort of 90 patients, with 45 in each group for analysis. The study was ethically approved by the Third Affiliated Hospital of Anhui Medical University (approval no. 2024-042-01).

### 2.2 Methods

#### 2.2.1 Establishment of SIRP

The establishment of SIRP consisted of several distinct phases: Phase 1 began with the immediate admission of patients through a dedicated “green channel,” where a specialized green channel nurse provided necessary examinations and preoperative care. In this phase, a specialized training group for patients with cerebral infarction was established, led by the department head and the head nurse, with the involvement of other medical staff. The team evaluated the motor function of each patient and developed a tailored rehabilitation training plan based on individual needs. Phase 2 focused on the creation of personalized training plans that aligned with the specific conditions and rehabilitation goals of the patients. The plans included a variety of exercises such as limb positioning, passive and active movements, sitting and balance training, walking training, muscle strength enhancement, joint range of motion exercises, and balance ability training. Phase 3 was dedicated to training the families or caregivers of the patients on how to assist with daily living activities. Phase 4 involved evaluating the home rehabilitation environment of the patient and developing a corresponding plan to ensure a smooth transition before discharge. Phase 5 emphasized regular follow-up through telephone calls or home visits to monitor the rehabilitation progress of the patients, adjust training plans as needed, and provide ongoing support and encouragement to both patients and their families.

A previous study that employed SIRP for patients with breast cancer focused on psycho-oncological interventions, which included psychological interventions, relaxation techniques, educational sessions, and various types of activating physiotherapy (9). In contrast, SIRP in this study adopted a holistic nursing model that involved physicians, nurses, patients, and family members and included long-term follow-up. The control group received standard nursing care, which included assessments, monitoring, routine observations of conditions, nursing documentation, psychological support, and health education. Meanwhile, the observation group received SIRP care in addition to standard care. This group benefited from a dedicated team that provided comprehensive treatment and support, enhancing the overall care framework. This approach aims to integrate and optimize healthcare delivery for better patient outcomes.

### 2.3 Evaluation indicators

#### 2.3.1 National Institute of Health Stroke Scale scores

The NIHSS scores were employed to evaluate the neurological deficits of the patients. A higher NIHSS score indicates more severe neurological impairments (12). The scale was validated in the Chinese population by Li et al. (13). The NIHSS includes various domains such as consciousness level, eye movements, muscle strength, sensation, coordination, language, and neglect, each scored on an ordinal scale. The total possible score ranges from 0 to 42, with higher scores indicating greater severity of the stroke.

#### 2.3.2 Barthel index evaluation

The evaluation of self-care ability was conducted using the Barthel Index, which comprises 10 components including eating, grooming, and mobility, with a total possible score of 100 points. A higher score on the Barthel Index indicates better self-care ability (14). The Barthel index was translated into Chinese by Gao et al. (15).

#### 2.3.3 Quality of life evaluation

The evaluation of quality of life is commonly conducted using the 36-item short-form survey (SF-36), an eight-dimensional, self-administered instrument designed to assess general health-related quality of life (HRQoL). The SF-36 includes eight domains: physical functioning (PF), role limitation due to physical problems (RP), bodily pain (BP), general health (GH), vitality (VT), social functioning (SF), role limitations due to emotional problems (RE), and mental health (MH). Scores for each domain range from 0 to 100, with higher scores indicating a better quality of life (16). The SF-36 scale was validated in Chinese by Ren et al. (17).

#### 2.3.4 Other indicators

In addition to questionnaires, other important indicators, such as complication identification and length of hospital stay, are considered when evaluating patient outcomes. Complications are identified based on the occurrence of specific postoperative events, including intracranial hemorrhage, upper gastrointestinal bleeding, reperfusion injury, lower extremity deep vein thrombosis, pulmonary infection, and subcutaneous hematoma at the puncture site. The length of hospital stay is determined by the duration between the admission and discharge of a patient, providing additional insights into the overall impact and efficiency of the treatment process.

#### 2.3.5 Statistical methods

Statistical analysis was performed using IBM SPSS Statistics for Windows (version 22.0; IBM Corp., Armonk, NY, United States). Data that followed a normal distribution were presented as mean ± standard deviation and compared between groups using the independent sample *t*-test. Data that followed a non-normal distribution were expressed as the median and interquartile range and analyzed using the Mann–Whitney U-test. Categorical data were presented as numbers (percentages) and analyzed using the chi-square test or Fisher’s exact test, whichever is appropriate. A significance level of *p* < 0.05 was considered to be statistically significant.

## 3 Results

### 3.1 Comparative analysis of general characteristics between the two patient groups

Among the 90 patients included in this study, 63 (70.0%) were male and 27 (30.0%) were female. There was no significant difference in the onset time between the two groups (5.23 ± 0.67 h vs. 5.29 ± 0.71 h; *p* = 0.671). The average age was 66.02 ± 8.86 years in the control group and 63.84 ± 8.74 years in the observation group, with the difference not reaching statistical significance (*p* > 0.05). Additionally, other baseline characteristics showed no significant differences between the groups, as detailed in Table 1.

**Table 1:** Comparison of general information between the two groups of patients.

	Observation group (*n* = 45)	Control group (*n* = 45)	*t* or χ^2^ value	*p* value
Age (years, *x* ± *s*)	63.84 ± 8.74	66.02 ± 8.86	−1.174	0.244
Gender (*n*, %)				
Male	31	32	0.053	0.818
Women	14	13		
Hypertension (*n*, %)	25	28	0.413	0.520
Diabetes mellitus (*n*, %)	10	8	0.278	0.598
Atrial fibrillation (*n*, %)	18	20	0.182	0.670
Time to onset (hours, *x* ± s)	5.23 ± 0.67	5.29 ± 0.71	−0.427	0.671
Site of vascular occlusion (*n*, %)				
Middle cerebral artery	21	23	1.359	0.507
Internal carotid artery	15	17		
Vertebrobasilar system	9	5		

### 3.2 Comparison of NIHSS scores between the two patient groups

The initial comparison of NIHSS scores upon admission showed no statistically significant differences between the two groups (*p* = 0.631), as detailed in Table 2. However, following SIRP, there was a notable difference in outcomes. One week postoperatively, patients in the observation group had significantly lower NIHSS scores compared to those in the control group (5.69 ± 1.72 vs. 12.62 ± 2.26; *p* < 0.001). This trend continued at 1 month (4.80 ± 0.99 vs. 11.24 ± 1.96; *p* < 0.001) and 3 months postoperatively (3.38 ± 1.19 vs. 10.24 ± 2.10; *p* < 0.001), indicating a substantial improvement in the observation group over time.

**Table 2:** Comparison of NIHSS scores between the two groups.

	NIHSS score (Points)			
	At the time of admission	1 week after surgery	1 month after surgery	3 months after surgery
Observation group (*n* = 45)	14.56 ± 2.19	5.69 ± 1.72	4.80 ± 0.99	3.38 ± 1.19
Control group (*n* = 45)	14.7 ± 2.18	12.62 ± 2.26	11.24 ± 1.96	10.24 ± 2.10
*t* value	−0.482	−16.392	−19.717	−19.064
*p* value	0.631	<0.001	<0.001	<0.001

### 3.3 Comparison of Barthel index between the two patient groups

Initially, there was no statistically significant difference in the Barthel index scores between the two patient groups upon admission (*p* = 0.799). However, following SIRP, significant differences emerged. Patients in the observation group showed significantly higher Barthel index scores compared to the control group at 1 week (34.96 ± 7.17 vs. 27.67 ± 5.04; *p* < 0.001), 1 month (53.93 ± 6.72 vs. 38.07 ± 4.93; *p* < 0.001), and 3 months postoperatively (62.49 ± 7.32 vs. 53.16 ± 4.37; *p* < 0.001). These outcomes are detailed in Table 3.

**Table 3:** Comparison of Barthel index between the two groups.

	Barthel index (Points)			
	At the time of admission	1 week after surgery	1 month after surgery	3 months after surgery
Observation group (*n* = 45)	22.09 ± 4.61	34.96 ± 7.17	53.93 ± 6.72	62.49 ± 7.32
Control group (*n* = 45)	22.36 ± 5.25	27.67 ± 5.04	38.07 ± 4.93	53.16 ± 4.37
*t* value	−0.256	5.580	12.764	7.343
*p* value	0.799	<0.001	<0.001	<0.001

### 3.4 Comparison of SF-36 between the two patient groups

**Figure 1:**
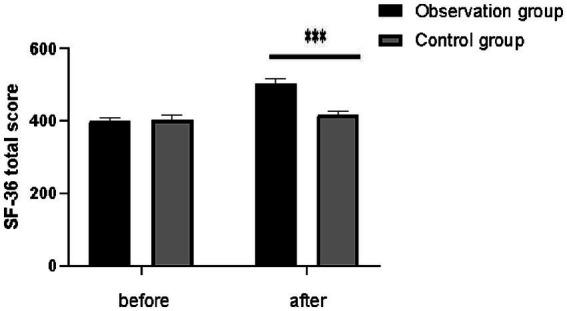
Comparison of SF-36 total score between the two groups. ^***^p < 0.001.

Initially, there were no significant differences in the SF-36 scores and its components between the observation and the control group scores (*p* > 0.05). However, significant improvements were observed by the three-month mark. The overall SF-36 scores were notably higher in the observation group compared to the control group (502.33 ± 14.28 vs. 417.64 ± 9.65, *p* < 0.001). Similarly, all the SF-36 subscores in the observation group were significantly better than those in the control group (all *p* < 0.05). Detailed results are shown in Table 4 and Figure 1.

**Table 4:** Comparison of SF-36 between the two groups at 3 months after surgery.

	Observation group (*n* = 45)	Control group (*n* = 45)	*t* value	*p* value
PF	65.26 ± 14.23	51.46 ± 12.28	7.286	<0.001
RP	60.40 ± 15.39	44.50 ± 10.69	8.049	<0.001
BP	61.38 ± 15.26	55.96 ± 18.42	4.368	0.003
GH	64.56 ± 14.99	56.38 ± 12.27	5.528	<0.001
VT	63.26 ± 14.44	52.16 ± 11.33	6.694	<0.001
SF	63.49 ± 12.19	49.87 ± 15.38	7.663	<0.001
RE	62.35 ± 13.28	51.02 ± 14.10	6.729	<0.001
MH	61.63 ± 15.58	56.38 ± 12.39	4.036	0.005
Total score	502.33 ± 14.28	417.64 ± 9.65	32.952	<0.001

PF, Physical functioning; RP, Physical problems; BP, Bodily pain; GH, General health; VT, Vitality; SF, Social functioning; RE, Role limitations due to emotional problems; and MH, Mental health.

### 3.5 Comparison of complication rates and length of stay between the two patient groups

**Figure 2:**
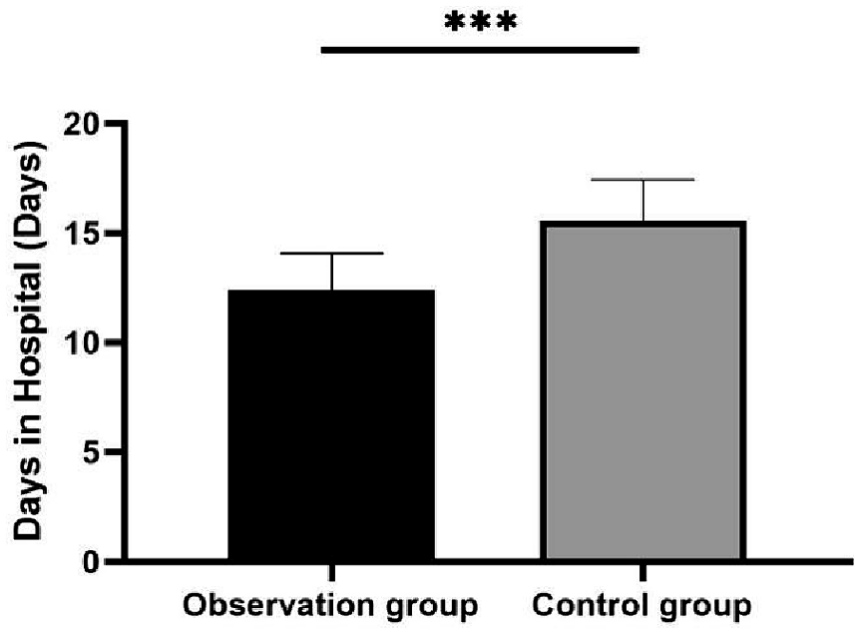
Comparison of hospital days between the two groups. ^***^p < 0.001.

The control group experienced an average hospital stay of 15.56 ± 1.87 days, whereas the observation group had a shorter average stay of 12.40 ± 1.68 days, significantly reducing the length of the hospital stay (*p* < 0.001; Figure 2). Regarding complications, the control group reported three cases of gastrointestinal bleeding, four cases of intracranial hemorrhage, two cases of reperfusion injury, three cases of pulmonary infection, and three cases of subcutaneous hematoma. In contrast, the observation group had only one case each of gastrointestinal bleeding, intracranial hemorrhage, and subcutaneous hematoma. The incidence of complications was significantly lower in the observation group than in the control group (*p* = 0.002). These findings are detailed in Table 5.

**Table 5:** Comparison of complication rates between the two groups [Cases (*n*)].

	Gastrointestinal bleeding	Intracranial hemorrhage	Reperfusion injury	Pulmonary infection	Subcutaneous hematoma	Total
Observation group (*n* = 45)	1	1	0	0	1	3
Control group (*n* = 45)	3	4	2	3	3	15
χ^2^						10.000
p						0.002

## 4 Discussion

Ischemic stroke is characterized by its sudden onset and rapid progression, which demands immediate reperfusion of the occluded vessel. Mechanical intravascular stent implantation, which involves inserting a thrombus stent to remove the occlusion, is a commonly employed and effective approach for this purpose (18, 19). However, patients who undergo intravascular stent implantation are at a heightened risk for various complications, such as intracranial hemorrhage, reperfusion injury, and infections. These complications are often exacerbated by compromised immunity and prolonged immobilization, potentially hindering recovery and increasing the risk of mortality (20, 21). Vigilant and proactive monitoring by nursing staff after the procedure is crucial in preventing deterioration, enhancing the success of the procedure, facilitating patient recovery, and ultimately improving the quality of life (22).

Step-by-step inpatient rehabilitation program is progressive reinforcement training, which refers to the method of gradually adapting and strengthening a particular skill or behavior through continuous and repeated training. Its primary goal is to provide high-quality nursing services, ensuring that patients receive effective care (23). In this study, patients who received SIRP following intravascular stent implantation showed significantly lower NIHSS scores and higher Barthel index scores at 1 week, 1 month, and 3 months postoperatively compared to the control group. Additionally, SIRP significantly enhanced the quality of life for patients with ACI who underwent intravascular stent implantation, aligning with the findings from the previous studies (9).

Research by Chen et al. (24) on integrated emergency nursing for patients with ACI showed that this approach could effectively reduce triage time and improve neurological function. Similarly, this study found that the observation group, which received SIRP, had a significantly shorter hospital stay and improved quality of life compared to the control group. These outcomes suggest that SIRP not only helps reduce the length of hospital stay, thus lessening the economic burden on families but also significantly enhances the neurological recovery and self-care abilities of patients with ischemic stroke following intravascular stent implantation, thereby enhancing their overall quality of life.

Previous research has shown that the overall complication rate associated with intravascular stent implantation ranges between 5 and 20% (25). The occurrence of complications after such procedures is critical in determining both the length of hospital stays and patient outcomes. Styczen et al. (26) conducted a retrospective analysis on patients who underwent mechanical intravascular stent implantation at seven tertiary care centers from January 2013 to May 2020, revealing a 19% incidence of postoperative symptomatic intracranial hemorrhage. At our institution, the implementation of SIRP, a specialized postoperative care protocol, has been effective in mitigating blood pressure elevation caused by patient anxiety or emotional distress. Through vigilant postoperative monitoring, any abnormal blood pressure fluctuations are promptly identified and managed, reducing the likelihood of postoperative intracranial hemorrhage and reperfusion injury. The study demonstrated that the incidence of intracranial hemorrhage was significantly lower in the observation group at 2.2%, compared to 8.9% in the control group, and there were no instances of reperfusion injury in the observation group, in contrast to 4.4% in the control group. These findings highlight the significant role of SIRP in reducing postoperative complications.

A previous study has shown that the implementation of standardized perioperative care protocols significantly enhances patient outcomes for those undergoing mechanical intravascular stent implantation for ACI (27). At our institution, SIRP was developed to provide consistent nursing interventions for individuals undergoing intravascular stent implantation for ACI. This comprehensive protocol includes preoperative preparation, postoperative management, rehabilitation exercises, psychological support, management of complications, and continuity of care. Its goal is to offer comprehensive and standardized nursing services to patients undergoing this procedure. Despite the widespread adoption of intravascular stent implantation across various healthcare facilities, postoperative recovery outcomes often remain suboptimal due to the lack of standardized care practices. Thus, it is crucial to apply scientifically sound and effective care strategies to patients undergoing vascular recanalization procedures. Implementing these strategies will maximize the efficacy of this technology and improve patient recovery and outcomes (28, 29).

The study still has certain limitations, including potential bias in sample selection. Additionally, being a single-center clinical trial, it highlights the need for further validation through multi-center clinical studies. The relatively small sample size also highlights the necessity of expanding it in future studies to enhance the credibility of the results. The occurrence of five cases of surgical failure and 11 cases of postoperative mortality within 3 months further complicates the assessment of the effectiveness of SIRP. Considering the distinct pathophysiology, prognosis, and clinical characteristics of lacunar stroke compared to other types of cerebral infarcts (30), future research could beneficially explore the impact of SIRP on lacunar versus non-lacunar ischemic strokes. Additionally, examining the effects on other subtypes of ischemic strokes, such as atherothrombotic, cardioembolic, unusual, and essential infarcts, could provide more comprehensive insights.

In this study, we observed that the use of SIRP, compared to conventional care approaches, resulted in significant improvements in neurological function recovery, self-care abilities, complication rates, length of hospital stays, quality of life, and overall postoperative recovery in patients with ACI who underwent intravascular stent implantation.

## Data availability statement

The raw data supporting the conclusions of this article will be made available by the authors, without undue reservation.

## Ethics statement

The study was ethically approved by the Third Affiliated Hospital of Anhui Medical University. The studies were conducted in accordance with the local legislation and institutional requirements. The participants provided their written informed consent to participate in this study.

## Author contributions

CW: Conceptualization, Data curation, Formal Analysis, Investigation, Methodology, Writing – original draft. NX: Conceptualization, Data curation, Investigation, Writing – original draft. JT: Data curation, Investigation, Writing – original draft. QC: Data curation, Investigation, Writing – original draft. QB: Conceptualization, Project administration, Supervision, Writing – review & editing.
